# Crystal structure of 4,4′-diethynylbiphen­yl

**DOI:** 10.1107/S2056989015011494

**Published:** 2015-06-20

**Authors:** Tei Tagg, C. John McAdam, Brian H. Robinson, Jim Simpson

**Affiliations:** a School of Fundamental Science, Universiti Malaysia Terengganu, 21030 Kuala Terengganu, Malaysia; b Department of Chemistry, University of Otago, PO Box 56, Dunedin, New Zealand

**Keywords:** crystal structure, 4,4′-diethynyl­biphen­yl, C—H⋯π(ring) inter­actions, C C—H⋯π contacts

## Abstract

The title compound, C_16_H_10_, crystallizes with four unique mol­ecules, designated 1–4, in the asymmetric unit of the monoclinic unit cell. None of the mol­ecules is planar, with the benzene rings of mol­ecules 1–4 inclined to one another at angles of 42.41 (4), 24.07 (6), 42.59 (4) and 46.88 (4)°, respectively. In the crystal, weak C—H⋯π(ring) interactions, augmented by even weaker C C—H⋯π(alkyne) contacts, generate a three-dimensional network structure with inter­linked columns of mol­ecules formed along the *c*-axis direction.

## Abstract 2

4,4′-Diethynylbiphenyl crystallizes with four unique mol­ecules in the asymmetric unit. The crystal structure is stabilized by weak C—H⋯π(ring) and C C—H⋯π(alkyne) contacts.

## Chemical context   

Donor–acceptor (*D*–*A*) dyads with the innate ability to generate long-lived charge separation in their excited states have elicited a great deal of current inter­est. Their applications cover fields ranging from artificial photosynthesis to solar cell technology (Rogozina *et al.*, 2013 ▸; Fukuzumi *et al.*, 2014 ▸). We have produced a variety of such dyads based on ferrocene as the donor and with a variety of acceptors (see for example: Flood *et al.*, 2007 ▸; Cuffe *et al.*, 2005 ▸; McAdam *et al.*, 2003 ▸). More recently, we have been inter­ested in expanding the range of donor–acceptor dyads by inter­polating a potentially conductive spacer between the donor and the acceptor to yield donor–spacer–acceptor (*D*–*S*–*A*) dyads. Biphenyl is a conductive spacer that we have used with some recent success, joined to a ferrocene donor through an alkene unit and to an acceptor *via* an alkyne link (McAdam *et al.*, 2010 ▸; Tagg *et al.*, 2015 ▸). We are inter­ested in further developing the chemistry of biphenyl as a potential spacer, with alkyne links to both the donor and the acceptor. Surprisingly, the mol­ecular and crystal structure of the precursor mol­ecule, 4,4′-diethynylbiphenyl (Liu, Liu *et al.*, 2005 ▸), has not been previously studied and we report its structure here.
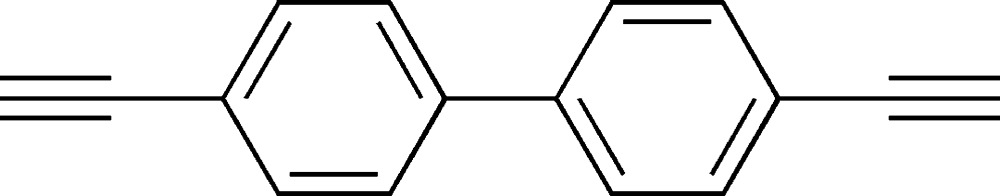


## Structural commentary   

**Figure 1:**
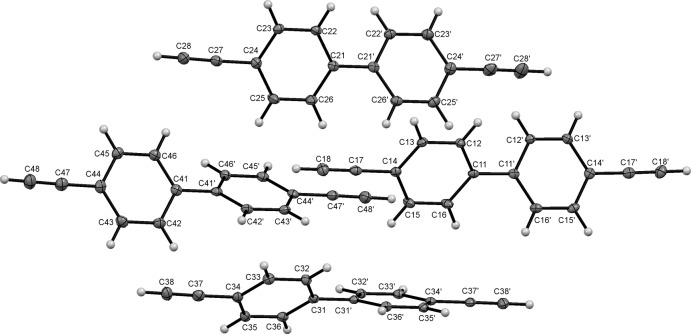
The asymmetric unit of (I), showing the numbering schemes for the four unique mol­ecules designated as types 1–4 with the types discriminated by the leading characters in the atom labels.

The title compound, (I), crystallizes with four unique mol­ecules in the asymmetric unit, identified by the leading digits 1–4 in the numbering schemes, Fig. 1 ▸. Each mol­ecule comprises a central biphenyl ring system symmetrically substituted at the 4 and 4′ positions by terminal alkyne units. None of the mol­ecules is planar, with the two benzene rings of each mol­ecule inclined to one another at angles of 42.41 (4), 24.07 (6), 42.59 (4) and 46.88 (4)° for mol­ecules 1–4, respectively. Bond distances and angles in the biphenyl ring systems are not unusual and compare well, both inter­nally, over the four unique mol­ecules, and with those observed in related systems (see for example: O’Brien *et al.*, 2010 ▸, Butler *et al.*, 2008 ▸; Muller, *et al.*, 2006 ▸, Nitsche *et al.*, 2003 ▸). The C*n*4—C*n*7 and C*n*4′—C*n*7′ distances (*n* = 1–4) [mean 1.445 (2) Å] are generally somewhat long, enough indeed to raise alerts in the *checkCIF* procedure. However analysis in *Vista* (Groom & Allen, 2014 ▸) of comparable values for eight other biphenyl systems, with terminal alkyne functions in the 4-position, provides a mean value of 1.442 (16) Å, not at all dissimilar to the values observed here (see for example: Langley *et al.*, 1998 ▸; Mague *et al.*, 1997 ▸; McAdam *et al.*, 2010 ▸; Laliberté *et al.*, 2006 ▸). The C C distances are also generally reasonable, with the exception of C27′—C28′, 1.130 (2) Å, which is unusually short compared to more typical C C distances of 1.181 (14) Å (Allen *et al.* 1987 ▸). There is no obvious explanation for this, except to note that the adjacent C27′—C24′ distance 1.4507 (19) Å is the longest of those reported here.

## Supra­molecular features   

The absence of donor and acceptor components, to provide classical hydrogen bonding or even C—H⋯*E* (*E* = O, N, halogen) contacts, challenge the packing in this system. There has been considerable speculation on the factors influencing the formation of structures with *Z*′ > 1 (Desiraju, 2007 ▸; Steed & Steed, 2015 ▸; Anderson & Steed 2007 ▸, Nichol & Clegg, 2007 ▸), and the nature, extent and degree of the inter­molecular contacts are clearly contributory factors. In this instance, the packing in the structure is profoundly influenced by an extensive series of weak edge-to-face C—H⋯π(ring) inter­actions (Table 1 ▸) augmented by still weaker C C—H⋯π(alkyne) contacts. It is likely that the inherent weakness of these contacts may influence the adoption of a *Z*′ > 1 structure.

**Figure 2:**
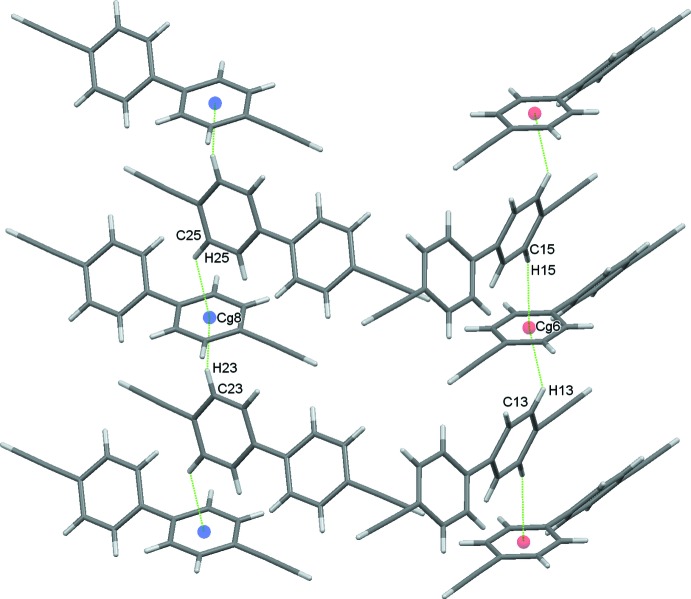
Complementary chains of 1, 3 and 2, 4 mol­ecules extending along the *c*-axis direction. In this and subsequent figures, C—H⋯π(ring) contacts are drawn as dotted lines with ring centroids shown as coloured spheres.

**Figure 3:**
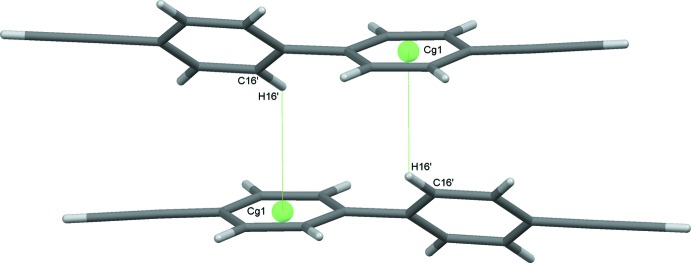
Inversion dimers formed through C—H⋯π(ring) contacts between mol­ecules of type 1.

**Figure 4:**
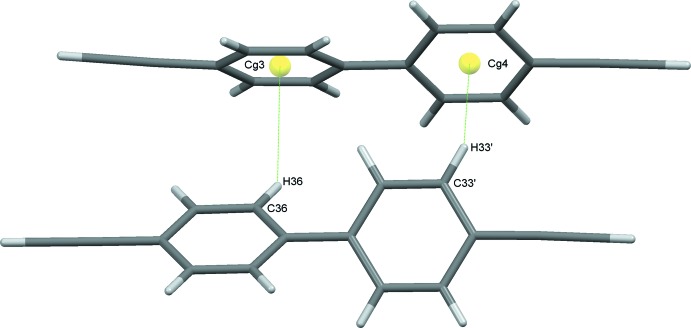
Dimers formed through C—H⋯π(ring) contacts between mol­ecules of types 2 and 4.

**Figure 5:**
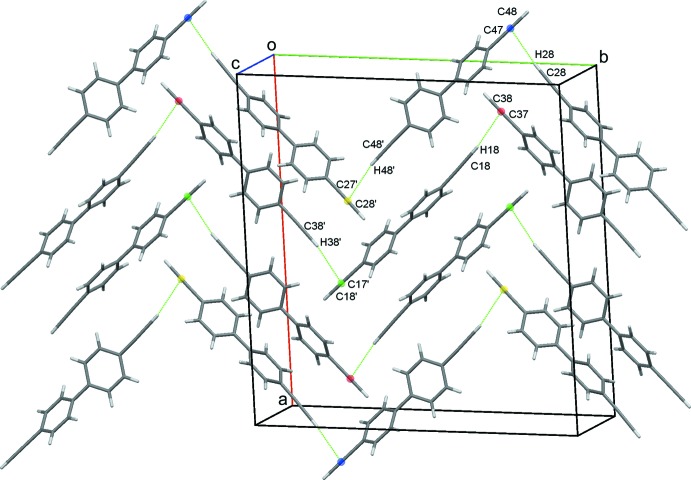
Zigzag chains of mol­ecules generated by C—H⋯C C contacts between mol­ecules of types 1 and 3 and mol­ecules of types 2 and 4. The centroids of the C C bonds are drawn as coloured spheres and the C—H⋯C C contacts are shown as dotted lines.

**Figure 6:**
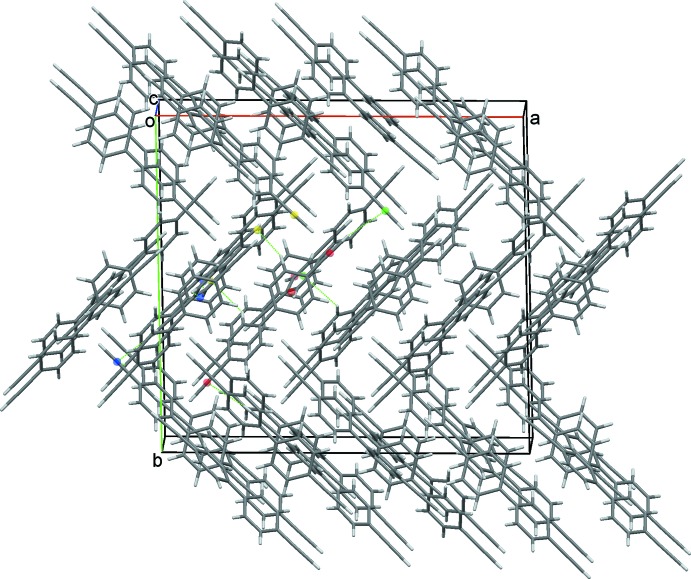
Overall packing for (I[Chem scheme1]) viewed along the *c* axis. Representative C—H⋯π(ring) and C—H⋯π(alkyne) contacts are drawn as dotted lines.

A complementary set of C—H⋯π contacts, involving in one case mol­ecules 1 and 3 and in the second mol­ecules 2 and 4, sandwiches a mol­ecule of 1 between two mol­ecules of 3 and a mol­ecule of 2 between two mol­ecules of 4. These contacts generate infinite chains approximately along the *c-*axis direction. The two chains lie approximately orthogonal to one another, Fig. 2 ▸. Weak C16′—H16′⋯*Cg*1 contacts form inversion dimers between two adjacent 1 mol­ecules, Fig. 3 ▸, and dimers also result from C—H⋯π contacts involving both rings of adjacent 2 and 3 mol­ecules, Fig. 4 ▸; both these sets of contacts contribute to the overall packing. In addition to these C—H⋯π(ring) inter­actions, one further set of somewhat unusual contacts is formed, again involving all four mol­ecules in the structure. These are weak C C—H⋯π(alkyne) contacts (Desiraju & Steiner, 1999 ▸) involving the relatively acidic C—H donors of the alkyne substituents. These again involve pairs of mol­ecules with C18—H18⋯C37 C38 and C38′—H38′⋯C17′ C18′ contacts generating one set of zigzag chains along *b* with an adjacent and complementary zigzag produced by C28—H28⋯C47 C48 and C48′—H48′⋯C27′ C28′ inter­actions, These chains generate layers of mol­ecules in the *ac* plane, Fig. 5 ▸. The contacts display the classic T shape, found also in the neutron structure of acetyl­ene (McMullan *et al.*, 1992 ▸), but not perfectly so. The H*n*8⋯C*n*7 distances are consistently slightly shorter [mean of the four distances = 2.77 (3) Å] than the H*n*8⋯C*n*8 equivalents [mean 2.97 (4) Å]. The mean H*n*8⋯C C centroid distance is 2.82 (4) Å and these values all fall well within projected ranges for such contacts (Desiraju & Steiner, 1999 ▸). The overall effect of this plethora of weak inter­actions is to stack mol­ecules into ‘multiple-decker sandwich’ columns, linked together along the *c*-axis direction, Fig. 6 ▸.

## Database survey   

Structures of 4-4′-disubstituted bi­phenyls abound with 2891 hits on the CSD (Groom & Allen, 2014 ▸). However, those with 4,4′-alkyne substituents are far less plentiful with only 29 entries. These fall into two distinct categories. First compounds with one or both of the alkyne substituents on the bi­phenyls bound to carbon or silicon atoms, 14 entries (see for example: Zhou *et al.*, 2012 ▸; McAdam *et al.*, 2010 ▸; O’Brien *et al.*, 2010 ▸, Zeng *et al.*, 2007 ▸; Muller, *et al.*, 2006 ▸; Nitsche *et al.*, 2003 ▸). Second, the well represented class of organometallic acetyl­ides, also referred to as ethynyl compounds. These have either the terminal hydrogen atoms of the alkyne groups both replaced by a transition metal complex moiety (see for example: Shanmugaraju *et al.*, 2011 ▸; Gao *et al.*, 2007 ▸; Ibn Ghazala *et al.*, 2006 ▸; Liu, Poon *et al.*, 2005 ▸) or, much less frequently, only a single terminal hydrogen atom is replaced to afford ethynyl complexes with terminal C C–H substituents (Zeng *et al.*, 2013 ▸; Saha *et al.*, 2005 ▸).

## Synthesis and crystallization   

The title compound (I) was prepared by a literature procedure (Liu, Liu *et al.*, 2005 ▸) and recrystallized from di­chloro­methane/hexane (1:1) to give pale-yellow plates suitable for X-ray analysis.

## Refinement   

Crystal data, data collection and structure refinement details are summarized in Table 2 ▸. All hydrogen atoms were refined using a riding model with *d*(C—H) = 0.95 Å, *U*_iso_ = 1.2*U*_eq_(C) for both the aromatic and terminal alkyne H atoms. Two low angle reflections with *F*_o_ << *F*_c_, with intensities likely to have been attenuated by the beam-stop, were removed for the final refinement cycles.

## Supplementary Material

Crystal structure: contains datablock(s) global, I. DOI: 10.1107/S2056989015011494/hg5446sup1.cif

Structure factors: contains datablock(s) I. DOI: 10.1107/S2056989015011494/hg5446Isup2.hkl

Supporting information file. DOI: 10.1107/S2056989015011494/hg5446Isup3.cml

CCDC reference: 1406589

Additional supporting information:  crystallographic information; 3D view; checkCIF report

## Figures and Tables

**Table 1:** CH interactions (, ) *Cg*1, *Cg*3, *Cg*4, *Cg*6 and *Cg*8 are the centroids of the C11C16, C21C26, C21C26, C31C36 and C41C46 rings, respectively.

*D*H*A*	*D*H	H*A*	*D* *A*	*D*H*A*
C13H13*Cg*6^i^	0.95	2.73	3.4910(13)	137
C15H15*Cg*6	0.95	2.70	3.4782(13)	140
C16H16*Cg*1^ii^	0.95	2.92	3.5375(12)	124
C23H23*Cg*8^i^	0.95	2.71	3.4809(13)	139
C25H25*Cg*8	0.95	2.76	3.4976(14)	136
C33H33*Cg*4^iii^	0.95	2.88	3.6153(13)	135
C36H36*Cg*3^iii^	0.95	2.87	3.6112(12)	135

Symmetry codes: (i) 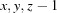; (ii) 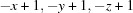; (iii) 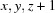.

**Table 2:** Experimental details

Crystal data	
Chemical formula	C_16_H_10_
*M*_r_	202.24
Crystal system, space group	Monoclinic, *P*2_1_/*c*
Temperature (K)	85
*a*, *b*, *c* ()	23.4263(5), 21.1181(5), 9.2989(2)
()	100.731(1)
*V* (^3^)	4519.89(17)
*Z*	16
Radiation type	Mo *K*
(mm^1^)	0.07
Crystal size (mm)	0.46 0.40 0.07

Data collection	
Diffractometer	BrukerNonius APEXII CCD
Absorption correction	Multi-scan (*SADABS*; Bruker, 2011 ▸)
*T*_min_, *T*_max_	0.887, 0.980
No. of measured, independent and observed [*I* > 2(*I*)] reflections	77658, 8885, 7147
*R*_int_	0.030
(sin /)_max_ (^1^)	0.617

Refinement	
*R*[*F* ^2^ > 2(*F* ^2^)], *wR*(*F* ^2^), *S*	0.036, 0.103, 1.03
No. of reflections	8885
No. of parameters	577
No. of restraints	42
H-atom treatment	H-atom parameters constrained
_max_, _min_ (e ^3^)	0.29, 0.16

Computer programs: , *APEX2* and *SAINT* (Bruker, 2011 ▸), *SHELXS* (Sheldrick, 2008 ▸), *SHELXL2014* (Sheldrick, 2015 ▸), *TITAN2000* (Hunter Simpson, 1999 ▸), *Mercury* (Macrae *et al.*, 2008 ▸), *enCIFer* (Allen *et al.*, 2004 ▸), *PLATON* (Spek, 2009 ▸) and *publCIF* (Westrip 2010 ▸).

## supplementary crystallographic information

### Crystal data

**Table:**

C_16_H_10_	*F*(000) = 1696
*M**_r_* = 202.24	*D*_x_ = 1.189 Mg m^−^^3^
Monoclinic, *P*2_1_/*c*	Mo *K*α radiation, λ = 0.71073 Å
*a* = 23.4263 (5) Å	Cell parameters from 8416 reflections
*b* = 21.1181 (5) Å	θ = 4.9–62.5°
*c* = 9.2989 (2) Å	µ = 0.07 mm^−^^1^
β = 100.731 (1)°	*T* = 85 K
*V* = 4519.89 (17) Å^3^	Plate, pale yellow
*Z* = 16	0.46 × 0.40 × 0.07 mm

### Data collection

**Table:**

Bruker–Nonius APEXII CCD diffractometer	8885 independent reflections
Radiation source: fine-focus sealed tube	7147 reflections with *I* > 2σ(*I*)
Graphite monochromator	*R*_int_ = 0.030
φ and ω scans	θ_max_ = 26.0°, θ_min_ = 1.3°
Absorption correction: multi-scan (*SADABS*; Bruker, 2011)	*h* = −28→28
*T*_min_ = 0.887, *T*_max_ = 0.980	*k* = −26→26
77658 measured reflections	*l* = −11→11

### Refinement

**Table:**

Refinement on *F*^2^	42 restraints
Least-squares matrix: full	Hydrogen site location: inferred from neighbouring sites
*R*[*F*^2^ > 2σ(*F*^2^)] = 0.036	H-atom parameters constrained
*wR*(*F*^2^) = 0.103	*w* = 1/[σ^2^(*F*_o_^2^) + (0.0471*P*)^2^ + 1.454*P*] where *P* = (*F*_o_^2^ + 2*F*_c_^2^)/3
*S* = 1.03	(Δ/σ)_max_ = 0.001
8885 reflections	Δρ_max_ = 0.29 e Å^−^^3^
577 parameters	Δρ_min_ = −0.16 e Å^−^^3^

### Special details

**Table:**

Geometry. All e.s.d.'s (except the e.s.d. in the dihedral angle between two l.s. planes) are estimated using the full covariance matrix. The cell e.s.d.'s are taken into account individually in the estimation of e.s.d.'s in distances, angles and torsion angles; correlations between e.s.d.'s in cell parameters are only used when they are defined by crystal symmetry. An approximate (isotropic) treatment of cell e.s.d.'s is used for estimating e.s.d.'s involving l.s. planes.
Refinement. Two low angle reflections with *F_o_* << Fc with intensities affected by the beam-stop were removed for the final refinement cycles.

### Fractional atomic coordinates and isotropic or equivalent isotropic displacement parameters (Å^2^)

**Table:**

	*x*	*y*	*z*	*U*_iso_*/*U*_eq_	
C18	0.26084 (6)	0.61801 (6)	0.27932 (15)	0.0318 (3)	
H18	0.2298	0.6477	0.2663	0.038*	
C17	0.29917 (5)	0.58137 (6)	0.29541 (13)	0.0240 (3)	
C16	0.40471 (6)	0.45861 (6)	0.46859 (13)	0.0252 (3)	
H16	0.4152	0.4381	0.5606	0.030*	
C15	0.36119 (5)	0.50352 (6)	0.44947 (13)	0.0253 (3)	
H15	0.3417	0.5130	0.5278	0.030*	
C14	0.34553 (5)	0.53520 (5)	0.31607 (13)	0.0206 (2)	
C13	0.37510 (5)	0.52063 (6)	0.20294 (13)	0.0226 (3)	
H13	0.3657	0.5423	0.1121	0.027*	
C12	0.41799 (5)	0.47484 (6)	0.22237 (12)	0.0221 (2)	
H12	0.4372	0.4650	0.1438	0.027*	
C11	0.43363 (5)	0.44272 (5)	0.35501 (12)	0.0196 (2)	
C11'	0.47811 (5)	0.39198 (5)	0.37373 (12)	0.0197 (2)	
C12'	0.47908 (5)	0.34774 (5)	0.26198 (12)	0.0214 (2)	
H12'	0.4516	0.3511	0.1732	0.026*	
C13'	0.51937 (5)	0.29931 (6)	0.27899 (13)	0.0229 (3)	
H13'	0.5192	0.2696	0.2023	0.027*	
C15'	0.56013 (5)	0.33806 (5)	0.52006 (13)	0.0231 (3)	
H15'	0.5880	0.3351	0.6082	0.028*	
C16'	0.51939 (5)	0.38625 (5)	0.50273 (12)	0.0222 (2)	
H16'	0.5195	0.4159	0.5796	0.027*	
C14'	0.56056 (5)	0.29371 (5)	0.40853 (13)	0.0212 (2)	
C17'	0.60326 (5)	0.24352 (6)	0.42461 (13)	0.0242 (3)	
C18'	0.63815 (6)	0.20297 (6)	0.43483 (14)	0.0294 (3)	
H18'	0.6663	0.1702	0.4431	0.035*	
C28	0.01222 (6)	0.61620 (7)	−0.10611 (15)	0.0359 (3)	
H28	−0.0189	0.6457	−0.1243	0.043*	
C27	0.05092 (6)	0.57953 (6)	−0.08355 (14)	0.0277 (3)	
C24	0.09698 (5)	0.53344 (6)	−0.05973 (13)	0.0231 (3)	
C25	0.12721 (6)	0.51937 (6)	0.08080 (14)	0.0292 (3)	
H25	0.1184	0.5417	0.1626	0.035*	
C26	0.16983 (6)	0.47332 (6)	0.10195 (13)	0.0280 (3)	
H26	0.1901	0.4647	0.1983	0.034*	
C23	0.11208 (5)	0.50095 (6)	−0.17817 (13)	0.0227 (3)	
H23	0.0931	0.5109	−0.2750	0.027*	
C22	0.15419 (5)	0.45468 (5)	−0.15572 (13)	0.0217 (2)	
H22	0.1634	0.4327	−0.2377	0.026*	
C21	0.18383 (5)	0.43917 (5)	−0.01531 (13)	0.0212 (2)	
C21'	0.22840 (5)	0.38863 (5)	0.00710 (13)	0.0219 (2)	
C22'	0.22770 (5)	0.33988 (6)	−0.09553 (13)	0.0247 (3)	
H22'	0.1977	0.3390	−0.1798	0.030*	
C23'	0.26965 (5)	0.29318 (6)	−0.07676 (14)	0.0278 (3)	
H23'	0.2686	0.2610	−0.1486	0.033*	
C25'	0.31418 (5)	0.34058 (6)	0.15210 (14)	0.0269 (3)	
H25'	0.3433	0.3405	0.2381	0.032*	
C26'	0.27264 (5)	0.38763 (6)	0.13152 (13)	0.0240 (3)	
H26'	0.2740	0.4200	0.2030	0.029*	
C24'	0.31377 (5)	0.29291 (6)	0.04756 (14)	0.0270 (3)	
C27'	0.35848 (6)	0.24449 (7)	0.06802 (16)	0.0352 (3)	
C28'	0.39403 (7)	0.20760 (7)	0.08536 (18)	0.0447 (4)	
H28'	0.4239	0.1766	0.0999	0.054*	
C38	0.11161 (6)	0.79841 (6)	0.69494 (16)	0.0334 (3)	
H38	0.0828	0.8306	0.6771	0.040*	
C37	0.14702 (5)	0.75884 (6)	0.71693 (14)	0.0274 (3)	
C34	0.19066 (5)	0.70967 (5)	0.74066 (13)	0.0231 (3)	
C35	0.18803 (5)	0.66086 (6)	0.84074 (13)	0.0240 (3)	
H35	0.1577	0.6605	0.8958	0.029*	
C36	0.22913 (5)	0.61314 (5)	0.86027 (13)	0.0218 (2)	
H36	0.2265	0.5800	0.9277	0.026*	
C33	0.23589 (5)	0.70960 (6)	0.66094 (13)	0.0250 (3)	
H33	0.2381	0.7423	0.5920	0.030*	
C32	0.27727 (5)	0.66226 (6)	0.68221 (13)	0.0229 (3)	
H32	0.3080	0.6631	0.6285	0.027*	
C31	0.27464 (5)	0.61318 (5)	0.78158 (12)	0.0199 (2)	
C31'	0.31938 (5)	0.56270 (5)	0.80312 (12)	0.0192 (2)	
C32'	0.30445 (5)	0.49923 (5)	0.81532 (12)	0.0209 (2)	
H32'	0.2648	0.4881	0.8105	0.025*	
C33'	0.34629 (5)	0.45206 (6)	0.83436 (12)	0.0220 (2)	
H33'	0.3352	0.4091	0.8416	0.026*	
C35'	0.41999 (5)	0.53127 (6)	0.83133 (12)	0.0225 (2)	
H35'	0.4597	0.5424	0.8375	0.027*	
C36'	0.37810 (5)	0.57790 (6)	0.81118 (12)	0.0211 (2)	
H36'	0.3892	0.6208	0.8027	0.025*	
C34'	0.40476 (5)	0.46768 (6)	0.84285 (12)	0.0206 (2)	
C37'	0.44936 (5)	0.41951 (6)	0.86414 (12)	0.0237 (3)	
C38'	0.48707 (6)	0.38135 (6)	0.88180 (14)	0.0302 (3)	
H38'	0.5173	0.3507	0.8960	0.036*	
C48	−0.13344 (6)	0.79702 (7)	0.30586 (17)	0.0402 (3)	
H48	−0.1621	0.8292	0.2858	0.048*	
C47	−0.09783 (6)	0.75706 (6)	0.33069 (15)	0.0324 (3)	
C44	−0.05457 (5)	0.70779 (6)	0.35722 (14)	0.0272 (3)	
C45	−0.05343 (5)	0.66035 (6)	0.25252 (14)	0.0264 (3)	
H45	−0.0811	0.6610	0.1637	0.032*	
C46	−0.01241 (5)	0.61263 (6)	0.27733 (13)	0.0241 (3)	
H46	−0.0127	0.5804	0.2060	0.029*	
C43	−0.01289 (6)	0.70624 (6)	0.48626 (15)	0.0297 (3)	
H43	−0.0132	0.7379	0.5586	0.036*	
C42	0.02873 (5)	0.65901 (6)	0.50944 (14)	0.0270 (3)	
H42	0.0572	0.6591	0.5968	0.032*	
C41	0.02949 (5)	0.61109 (6)	0.40583 (13)	0.0229 (3)	
C41'	0.07433 (5)	0.56078 (6)	0.42969 (12)	0.0219 (2)	
C42'	0.13294 (5)	0.57601 (6)	0.47849 (13)	0.0232 (3)	
H42'	0.1439	0.6189	0.4983	0.028*	
C43'	0.17498 (5)	0.52946 (6)	0.49820 (13)	0.0243 (3)	
H43'	0.2146	0.5407	0.5293	0.029*	
C45'	0.10123 (5)	0.45009 (6)	0.42502 (13)	0.0246 (3)	
H45'	0.0902	0.4071	0.4081	0.030*	
C46'	0.05956 (5)	0.49710 (6)	0.40248 (13)	0.0238 (3)	
H46'	0.0201	0.4860	0.3678	0.029*	
C44'	0.15977 (5)	0.46576 (6)	0.47284 (12)	0.0235 (3)	
C47'	0.20428 (5)	0.41762 (6)	0.49536 (13)	0.0273 (3)	
C48'	0.24191 (6)	0.37986 (7)	0.51539 (15)	0.0350 (3)	
H48'	0.2723	0.3494	0.5316	0.042*	

### Atomic displacement parameters (Å^2^)

**Table:**

	*U*^11^	*U*^22^	*U*^33^	*U*^12^	*U*^13^	*U*^23^
C18	0.0334 (7)	0.0309 (7)	0.0326 (7)	0.0041 (6)	0.0103 (6)	0.0050 (6)
C17	0.0262 (6)	0.0268 (6)	0.0198 (6)	−0.0057 (5)	0.0062 (5)	−0.0005 (5)
C16	0.0370 (7)	0.0220 (6)	0.0173 (6)	0.0024 (5)	0.0065 (5)	0.0022 (5)
C15	0.0341 (7)	0.0238 (6)	0.0201 (6)	0.0014 (5)	0.0105 (5)	−0.0016 (5)
C14	0.0213 (6)	0.0183 (5)	0.0217 (6)	−0.0030 (4)	0.0029 (5)	−0.0019 (4)
C13	0.0224 (6)	0.0276 (6)	0.0167 (6)	−0.0015 (5)	0.0011 (4)	0.0023 (5)
C12	0.0215 (6)	0.0288 (6)	0.0165 (6)	−0.0005 (5)	0.0049 (4)	−0.0005 (5)
C11	0.0216 (6)	0.0191 (6)	0.0175 (6)	−0.0044 (5)	0.0027 (4)	−0.0024 (4)
C11'	0.0213 (6)	0.0200 (6)	0.0186 (6)	−0.0041 (5)	0.0056 (4)	0.0019 (4)
C12'	0.0213 (6)	0.0251 (6)	0.0173 (6)	−0.0016 (5)	0.0023 (4)	−0.0001 (5)
C13'	0.0251 (6)	0.0231 (6)	0.0212 (6)	−0.0023 (5)	0.0062 (5)	−0.0031 (5)
C15'	0.0248 (6)	0.0234 (6)	0.0196 (6)	−0.0051 (5)	0.0001 (5)	0.0040 (5)
C16'	0.0282 (6)	0.0212 (6)	0.0171 (6)	−0.0036 (5)	0.0035 (5)	−0.0008 (4)
C14'	0.0205 (6)	0.0199 (6)	0.0238 (6)	−0.0033 (5)	0.0055 (5)	0.0040 (5)
C17'	0.0251 (6)	0.0244 (6)	0.0235 (6)	−0.0072 (5)	0.0057 (5)	0.0016 (5)
C18'	0.0274 (7)	0.0271 (7)	0.0332 (7)	−0.0001 (6)	0.0040 (5)	0.0067 (5)
C28	0.0371 (8)	0.0381 (8)	0.0313 (7)	0.0090 (6)	0.0027 (6)	−0.0049 (6)
C27	0.0301 (7)	0.0304 (7)	0.0230 (6)	−0.0038 (6)	0.0059 (5)	−0.0044 (5)
C24	0.0226 (6)	0.0218 (6)	0.0255 (6)	−0.0029 (5)	0.0060 (5)	−0.0006 (5)
C25	0.0363 (7)	0.0318 (7)	0.0215 (6)	0.0038 (6)	0.0102 (5)	−0.0031 (5)
C26	0.0340 (7)	0.0316 (7)	0.0186 (6)	0.0034 (6)	0.0052 (5)	0.0021 (5)
C23	0.0207 (6)	0.0264 (6)	0.0208 (6)	−0.0054 (5)	0.0033 (5)	−0.0003 (5)
C22	0.0215 (6)	0.0242 (6)	0.0201 (6)	−0.0050 (5)	0.0056 (5)	−0.0034 (5)
C21	0.0216 (6)	0.0214 (6)	0.0219 (6)	−0.0058 (5)	0.0071 (5)	0.0005 (5)
C21'	0.0228 (6)	0.0224 (6)	0.0219 (6)	−0.0054 (5)	0.0074 (5)	0.0018 (5)
C22'	0.0246 (6)	0.0253 (6)	0.0236 (6)	−0.0029 (5)	0.0026 (5)	−0.0011 (5)
C23'	0.0295 (7)	0.0240 (6)	0.0294 (7)	−0.0028 (5)	0.0040 (5)	−0.0046 (5)
C25'	0.0259 (6)	0.0271 (6)	0.0262 (6)	−0.0045 (5)	0.0006 (5)	0.0016 (5)
C26'	0.0271 (6)	0.0230 (6)	0.0223 (6)	−0.0048 (5)	0.0055 (5)	−0.0021 (5)
C24'	0.0252 (6)	0.0215 (6)	0.0336 (7)	−0.0021 (5)	0.0039 (5)	0.0005 (5)
C27'	0.0338 (7)	0.0306 (7)	0.0380 (8)	−0.0095 (6)	−0.0016 (6)	−0.0062 (6)
C28'	0.0388 (8)	0.0274 (7)	0.0606 (10)	0.0002 (7)	−0.0095 (7)	−0.0131 (7)
C38	0.0267 (7)	0.0301 (7)	0.0433 (8)	0.0003 (6)	0.0065 (6)	−0.0079 (6)
C37	0.0266 (6)	0.0254 (6)	0.0298 (7)	−0.0073 (5)	0.0043 (5)	−0.0049 (5)
C34	0.0206 (6)	0.0208 (6)	0.0265 (6)	−0.0021 (5)	0.0011 (5)	−0.0059 (5)
C35	0.0208 (6)	0.0262 (6)	0.0259 (6)	−0.0041 (5)	0.0069 (5)	−0.0061 (5)
C36	0.0231 (6)	0.0218 (6)	0.0208 (6)	−0.0048 (5)	0.0043 (5)	−0.0016 (5)
C33	0.0264 (6)	0.0231 (6)	0.0254 (6)	−0.0023 (5)	0.0044 (5)	0.0013 (5)
C32	0.0215 (6)	0.0249 (6)	0.0232 (6)	−0.0027 (5)	0.0068 (5)	−0.0006 (5)
C31	0.0193 (5)	0.0210 (6)	0.0188 (6)	−0.0041 (5)	0.0018 (4)	−0.0043 (4)
C31'	0.0202 (6)	0.0246 (6)	0.0130 (5)	−0.0022 (5)	0.0035 (4)	−0.0009 (4)
C32'	0.0190 (5)	0.0259 (6)	0.0181 (6)	−0.0037 (5)	0.0042 (4)	−0.0005 (5)
C33'	0.0258 (6)	0.0224 (6)	0.0178 (6)	−0.0028 (5)	0.0039 (5)	0.0003 (5)
C35'	0.0193 (6)	0.0300 (6)	0.0183 (6)	−0.0026 (5)	0.0039 (4)	−0.0004 (5)
C36'	0.0221 (6)	0.0220 (6)	0.0196 (6)	−0.0045 (5)	0.0046 (5)	−0.0004 (5)
C34'	0.0229 (6)	0.0267 (6)	0.0123 (5)	0.0017 (5)	0.0031 (4)	−0.0004 (4)
C37'	0.0253 (6)	0.0303 (7)	0.0154 (6)	−0.0024 (5)	0.0037 (5)	−0.0008 (5)
C38'	0.0328 (7)	0.0335 (7)	0.0236 (6)	0.0073 (6)	0.0041 (5)	0.0007 (5)
C48	0.0328 (8)	0.0351 (8)	0.0517 (9)	0.0024 (6)	0.0056 (7)	−0.0063 (7)
C47	0.0305 (7)	0.0281 (7)	0.0381 (8)	−0.0055 (6)	0.0048 (6)	−0.0034 (6)
C44	0.0239 (6)	0.0240 (6)	0.0346 (7)	−0.0037 (5)	0.0075 (5)	0.0007 (5)
C45	0.0227 (6)	0.0272 (6)	0.0288 (7)	−0.0063 (5)	0.0034 (5)	0.0004 (5)
C46	0.0237 (6)	0.0239 (6)	0.0256 (6)	−0.0071 (5)	0.0071 (5)	−0.0033 (5)
C43	0.0320 (7)	0.0257 (7)	0.0319 (7)	−0.0025 (5)	0.0076 (6)	−0.0062 (5)
C42	0.0289 (6)	0.0278 (6)	0.0233 (6)	−0.0037 (5)	0.0028 (5)	−0.0025 (5)
C41	0.0222 (6)	0.0226 (6)	0.0252 (6)	−0.0062 (5)	0.0079 (5)	0.0001 (5)
C41'	0.0247 (6)	0.0260 (6)	0.0160 (5)	−0.0046 (5)	0.0061 (5)	−0.0013 (5)
C42'	0.0265 (6)	0.0242 (6)	0.0191 (6)	−0.0052 (5)	0.0042 (5)	−0.0019 (5)
C43'	0.0231 (6)	0.0323 (7)	0.0173 (6)	−0.0058 (5)	0.0030 (5)	−0.0017 (5)
C45'	0.0285 (6)	0.0242 (6)	0.0224 (6)	−0.0048 (5)	0.0080 (5)	−0.0025 (5)
C46'	0.0220 (6)	0.0271 (6)	0.0229 (6)	−0.0045 (5)	0.0061 (5)	−0.0024 (5)
C44'	0.0269 (6)	0.0291 (6)	0.0153 (6)	0.0006 (5)	0.0060 (5)	−0.0006 (5)
C47'	0.0293 (7)	0.0333 (7)	0.0192 (6)	−0.0042 (6)	0.0046 (5)	−0.0034 (5)
C48'	0.0377 (8)	0.0360 (8)	0.0300 (7)	0.0065 (6)	0.0032 (6)	−0.0031 (6)

### Geometric parameters (Å, º)

**Table:**

C18—C17	1.1736 (18)	C38—C37	1.1684 (18)
C18—H18	0.9500	C38—H38	0.9500
C17—C14	1.4454 (17)	C37—C34	1.4450 (17)
C16—C15	1.3795 (17)	C34—C35	1.3977 (17)
C16—C11	1.3977 (16)	C34—C33	1.4018 (17)
C16—H16	0.9500	C35—C36	1.3823 (17)
C15—C14	1.3968 (17)	C35—H35	0.9500
C15—H15	0.9500	C36—C31	1.4009 (16)
C14—C13	1.3974 (16)	C36—H36	0.9500
C13—C12	1.3820 (17)	C33—C32	1.3809 (17)
C13—H13	0.9500	C33—H33	0.9500
C12—C11	1.3955 (16)	C32—C31	1.3976 (16)
C12—H12	0.9500	C32—H32	0.9500
C11—C11'	1.4821 (16)	C31—C31'	1.4820 (16)
C11'—C16'	1.3988 (16)	C31'—C32'	1.3953 (16)
C11'—C12'	1.4008 (16)	C31'—C36'	1.4009 (15)
C12'—C13'	1.3806 (16)	C32'—C33'	1.3854 (16)
C12'—H12'	0.9500	C32'—H32'	0.9500
C13'—C14'	1.4011 (17)	C33'—C34'	1.3968 (16)
C13'—H13'	0.9500	C33'—H33'	0.9500
C15'—C16'	1.3839 (17)	C35'—C36'	1.3783 (16)
C15'—C14'	1.3989 (17)	C35'—C34'	1.3986 (16)
C15'—H15'	0.9500	C35'—H35'	0.9500
C16'—H16'	0.9500	C36'—H36'	0.9500
C14'—C17'	1.4459 (17)	C34'—C37'	1.4452 (17)
C17'—C18'	1.1753 (18)	C37'—C38'	1.1843 (18)
C18'—H18'	0.9500	C38'—H38'	0.9500
C28—C27	1.1810 (19)	C48—C47	1.179 (2)
C28—H28	0.9500	C48—H48	0.9500
C27—C24	1.4394 (17)	C47—C44	1.4413 (18)
C24—C25	1.3974 (17)	C44—C43	1.3993 (18)
C24—C23	1.3979 (17)	C44—C45	1.4008 (18)
C25—C26	1.3815 (18)	C45—C46	1.3820 (17)
C25—H25	0.9500	C45—H45	0.9500
C26—C21	1.3963 (17)	C46—C41	1.3984 (17)
C26—H26	0.9500	C46—H46	0.9500
C23—C22	1.3765 (17)	C43—C42	1.3834 (18)
C23—H23	0.9500	C43—H43	0.9500
C22—C21	1.3988 (16)	C42—C41	1.3997 (17)
C22—H22	0.9500	C42—H42	0.9500
C21—C21'	1.4803 (17)	C41—C41'	1.4814 (17)
C21'—C22'	1.4019 (17)	C41'—C46'	1.3998 (17)
C21'—C26'	1.4023 (17)	C41'—C42'	1.4009 (16)
C22'—C23'	1.3803 (17)	C42'—C43'	1.3793 (17)
C22'—H22'	0.9500	C42'—H42'	0.9500
C23'—C24'	1.3999 (18)	C43'—C44'	1.4003 (17)
C23'—H23'	0.9500	C43'—H43'	0.9500
C25'—C26'	1.3790 (17)	C45'—C46'	1.3806 (17)
C25'—C24'	1.3982 (18)	C45'—C44'	1.4004 (17)
C25'—H25'	0.9500	C45'—H45'	0.9500
C26'—H26'	0.9500	C46'—H46'	0.9500
C24'—C27'	1.4507 (19)	C44'—C47'	1.4433 (18)
C27'—C28'	1.130 (2)	C47'—C48'	1.1776 (19)
C28'—H28'	0.9500	C48'—H48'	0.9500
			
C17—C18—H18	180.0	C37—C38—H38	180.0
C18—C17—C14	178.75 (13)	C38—C37—C34	178.73 (14)
C15—C16—C11	121.21 (11)	C35—C34—C33	118.87 (11)
C15—C16—H16	119.4	C35—C34—C37	120.93 (11)
C11—C16—H16	119.4	C33—C34—C37	120.19 (11)
C16—C15—C14	120.62 (11)	C36—C35—C34	120.60 (11)
C16—C15—H15	119.7	C36—C35—H35	119.7
C14—C15—H15	119.7	C34—C35—H35	119.7
C15—C14—C13	118.56 (11)	C35—C36—C31	120.68 (11)
C15—C14—C17	120.51 (11)	C35—C36—H36	119.7
C13—C14—C17	120.93 (10)	C31—C36—H36	119.7
C12—C13—C14	120.40 (11)	C32—C33—C34	120.30 (11)
C12—C13—H13	119.8	C32—C33—H33	119.8
C14—C13—H13	119.8	C34—C33—H33	119.8
C13—C12—C11	121.36 (11)	C33—C32—C31	121.03 (11)
C13—C12—H12	119.3	C33—C32—H32	119.5
C11—C12—H12	119.3	C31—C32—H32	119.5
C12—C11—C16	117.83 (11)	C32—C31—C36	118.51 (11)
C12—C11—C11'	121.22 (10)	C32—C31—C31'	120.38 (10)
C16—C11—C11'	120.92 (10)	C36—C31—C31'	121.11 (10)
C16'—C11'—C12'	118.31 (11)	C32'—C31'—C36'	118.30 (11)
C16'—C11'—C11	121.46 (10)	C32'—C31'—C31	121.36 (10)
C12'—C11'—C11	120.22 (10)	C36'—C31'—C31	120.34 (10)
C13'—C12'—C11'	120.95 (11)	C33'—C32'—C31'	121.27 (11)
C13'—C12'—H12'	119.5	C33'—C32'—H32'	119.4
C11'—C12'—H12'	119.5	C31'—C32'—H32'	119.4
C12'—C13'—C14'	120.52 (11)	C32'—C33'—C34'	120.02 (11)
C12'—C13'—H13'	119.7	C32'—C33'—H33'	120.0
C14'—C13'—H13'	119.7	C34'—C33'—H33'	120.0
C16'—C15'—C14'	120.40 (11)	C36'—C35'—C34'	120.73 (11)
C16'—C15'—H15'	119.8	C36'—C35'—H35'	119.6
C14'—C15'—H15'	119.8	C34'—C35'—H35'	119.6
C15'—C16'—C11'	121.01 (11)	C35'—C36'—C31'	120.72 (11)
C15'—C16'—H16'	119.5	C35'—C36'—H36'	119.6
C11'—C16'—H16'	119.5	C31'—C36'—H36'	119.6
C15'—C14'—C13'	118.81 (11)	C33'—C34'—C35'	118.94 (11)
C15'—C14'—C17'	120.96 (11)	C33'—C34'—C37'	121.18 (11)
C13'—C14'—C17'	120.22 (11)	C35'—C34'—C37'	119.87 (10)
C18'—C17'—C14'	178.63 (13)	C38'—C37'—C34'	178.12 (13)
C17'—C18'—H18'	180.0	C37'—C38'—H38'	180.0
C27—C28—H28	180.0	C47—C48—H48	180.0
C28—C27—C24	178.05 (14)	C48—C47—C44	178.56 (15)
C25—C24—C23	118.29 (11)	C43—C44—C45	118.61 (11)
C25—C24—C27	121.50 (11)	C43—C44—C47	121.32 (12)
C23—C24—C27	120.21 (11)	C45—C44—C47	120.08 (12)
C26—C25—C24	120.65 (11)	C46—C45—C44	120.54 (12)
C26—C25—H25	119.7	C46—C45—H45	119.7
C24—C25—H25	119.7	C44—C45—H45	119.7
C25—C26—C21	121.40 (11)	C45—C46—C41	121.01 (11)
C25—C26—H26	119.3	C45—C46—H46	119.5
C21—C26—H26	119.3	C41—C46—H46	119.5
C22—C23—C24	120.55 (11)	C42—C43—C44	120.63 (12)
C22—C23—H23	119.7	C42—C43—H43	119.7
C24—C23—H23	119.7	C44—C43—H43	119.7
C23—C22—C21	121.67 (11)	C43—C42—C41	120.86 (12)
C23—C22—H22	119.2	C43—C42—H42	119.6
C21—C22—H22	119.2	C41—C42—H42	119.6
C26—C21—C22	117.39 (11)	C46—C41—C42	118.33 (11)
C26—C21—C21'	121.63 (11)	C46—C41—C41'	120.66 (11)
C22—C21—C21'	120.98 (11)	C42—C41—C41'	120.99 (11)
C22'—C21'—C26'	117.62 (11)	C46'—C41'—C42'	118.25 (11)
C22'—C21'—C21	121.01 (11)	C46'—C41'—C41	121.16 (10)
C26'—C21'—C21	121.37 (11)	C42'—C41'—C41	120.58 (11)
C23'—C22'—C21'	121.39 (11)	C43'—C42'—C41'	120.79 (11)
C23'—C22'—H22'	119.3	C43'—C42'—H42'	119.6
C21'—C22'—H22'	119.3	C41'—C42'—H42'	119.6
C22'—C23'—C24'	120.41 (12)	C42'—C43'—C44'	120.62 (11)
C22'—C23'—H23'	119.8	C42'—C43'—H43'	119.7
C24'—C23'—H23'	119.8	C44'—C43'—H43'	119.7
C26'—C25'—C24'	120.59 (11)	C46'—C45'—C44'	120.07 (11)
C26'—C25'—H25'	119.7	C46'—C45'—H45'	120.0
C24'—C25'—H25'	119.7	C44'—C45'—H45'	120.0
C25'—C26'—C21'	121.30 (11)	C45'—C46'—C41'	121.30 (11)
C25'—C26'—H26'	119.4	C45'—C46'—H46'	119.3
C21'—C26'—H26'	119.4	C41'—C46'—H46'	119.3
C25'—C24'—C23'	118.66 (11)	C43'—C44'—C45'	118.94 (11)
C25'—C24'—C27'	120.33 (12)	C43'—C44'—C47'	119.87 (11)
C23'—C24'—C27'	121.01 (12)	C45'—C44'—C47'	121.19 (11)
C28'—C27'—C24'	178.68 (15)	C48'—C47'—C44'	177.80 (14)
C27'—C28'—H28'	180.0	C47'—C48'—H48'	180.0

### Hydrogen-bond geometry (Å, º)

Cg1, Cg3, Cg4, Cg6 and Cg8 are the centroids of the C11–C16, C21–C26, C21'–C26', C31'–C36' and C41'–C46' rings, respectively.

**Table:**

*D*—H···*A*	*D*—H	H···*A*	*D*···*A*	*D*—H···*A*
C13—H13···*Cg*6^i^	0.95	2.73	3.4910 (13)	137
C15—H15···*Cg*6	0.95	2.70	3.4782 (13)	140
C16′—H16′···*Cg*1^ii^	0.95	2.92	3.5375 (12)	124
C23—H23···*Cg*8^i^	0.95	2.71	3.4809 (13)	139
C25—H25···*Cg*8	0.95	2.76	3.4976 (14)	136
C33′—H33′···*Cg*4^iii^	0.95	2.88	3.6153 (13)	135
C36—H36···*Cg*3^iii^	0.95	2.87	3.6112 (12)	135

Symmetry codes: (i) *x*, *y*, *z*−1; (ii) −*x*+1, −*y*+1, −*z*+1; (iii) *x*, *y*, *z*+1.
